# Retropharyngeal Ectopic Thymus in a Pediatric Patient With 22q11.2 Deletion Syndrome

**DOI:** 10.7759/cureus.33350

**Published:** 2023-01-04

**Authors:** Kacper Grudzień, Julia Kuzaj, Magdalena Dębicka, Stanisław Kwiatkowski, Olga Milczarek

**Affiliations:** 1 Department of Neurosurgery, University Children's Hospital, Kraków, POL; 2 Department of Neurosurgery and Neurotraumatology, The University Hospital, Kraków, POL

**Keywords:** 22q11.2 deletion, digeorge, thymus, ectopic, retropharyngeal

## Abstract

The thymus gland plays a crucial role in the maturation process of lymphocyte T cells. Developmental disorders of this organ might be caused by genetic diseases, such as the 22q11.2 deletion and DiGeorge syndrome. Other manifestations of this condition are heart defects, a reduced number of T cells, hypocalcemia, and facial dysmorphia.

A 13-year-old boy with 22q11 deletion syndrome presented with paresis and paresthesia of the right upper extremity. Magnetic resonance imaging (MRI) revealed a solid mass in the retropharyngeal and prevertebral areas. The lesion was excised and, upon histopathological examination, turned out to be ectopic thymic tissue. A follow-up examination showed no recurrence of the lesion.

The ectopic thymus is a rare pathology, especially in 22q11 deletion syndrome patients. In general, thymic tissue can be found anywhere along its normal path of descent. In this case, however, its location cannot be explained solely by its embryological origin, as at no point should the thymus or its histological predecessor be located in the retropharyngeal area. As such, this finding challenges our current understanding of thymic embryological genesis.

## Introduction

The thymus gland is a bilobed organ located in the anterosuperior mediastinum. It plays a significant role in the immune system, providing an appropriate environment for the maturation process of T cells [1]. Embryologically, the thymus originates bilaterally from the third laryngeal pouch in the seventh week of gestation, travels anteriorly and caudally, and eventually fuses at the top of the pericardium in the mid-eighth week [2,3]. Defective pathways of embryological development may lead to a clinical spectrum of anomalies. Absence, hypoplasia, or ectopia of the thymus may be caused by genetic disorders, such as the 22q11.2 deletion syndrome. This condition may also clinically present as heart defects, CD3^+^ lymphocyte T count less than 500/mm^3^, hypocalcemia, neuropsychiatric disorders, cleft palate, and facial dysmorphia [4]. One rare abnormality of the thymus is ectopia, which accounts for 2.3% of findings in children scheduled for an ultrasonographic evaluation [5]. This finding is particularly unusual in 22q11 deletion syndrome, as the usual pathology associated with this condition is thymic hypoplasia, aplasia, or ectopia along the normal path of its descent.

In this study, we aim to highlight the importance of considering the ectopic thymus in the differential diagnosis of neck masses.

This article was previously presented as a meeting abstract at the 2022 International Medical Students' Conference on June 3, 2022, in Kraków.

## Case presentation

A 13-year-old boy with 22q11.2 deletion syndrome (46 XY, del 22q11.2) was admitted due to numbness of the upper right extremity, dysphagia, and dysarthria that had lasted for four months. Physical examination showed a keeled chest, mild lateral spinal curvature, and winged scapulae. He presented face dysmorphia (broad nose base, epicanthus, micro- and retrognathism), height deficiency (−2.0 SD), and low body mass (weight-to-height ratio −20%). No characteristic features of 22q11.2 deletion syndrome were found: lymphocytes T CD3^+^ 1860/µl, Ca^2+^ 2.45 mmol/l, no heart defects except irrelevant tricuspid incompetence.

**Figure 1:**
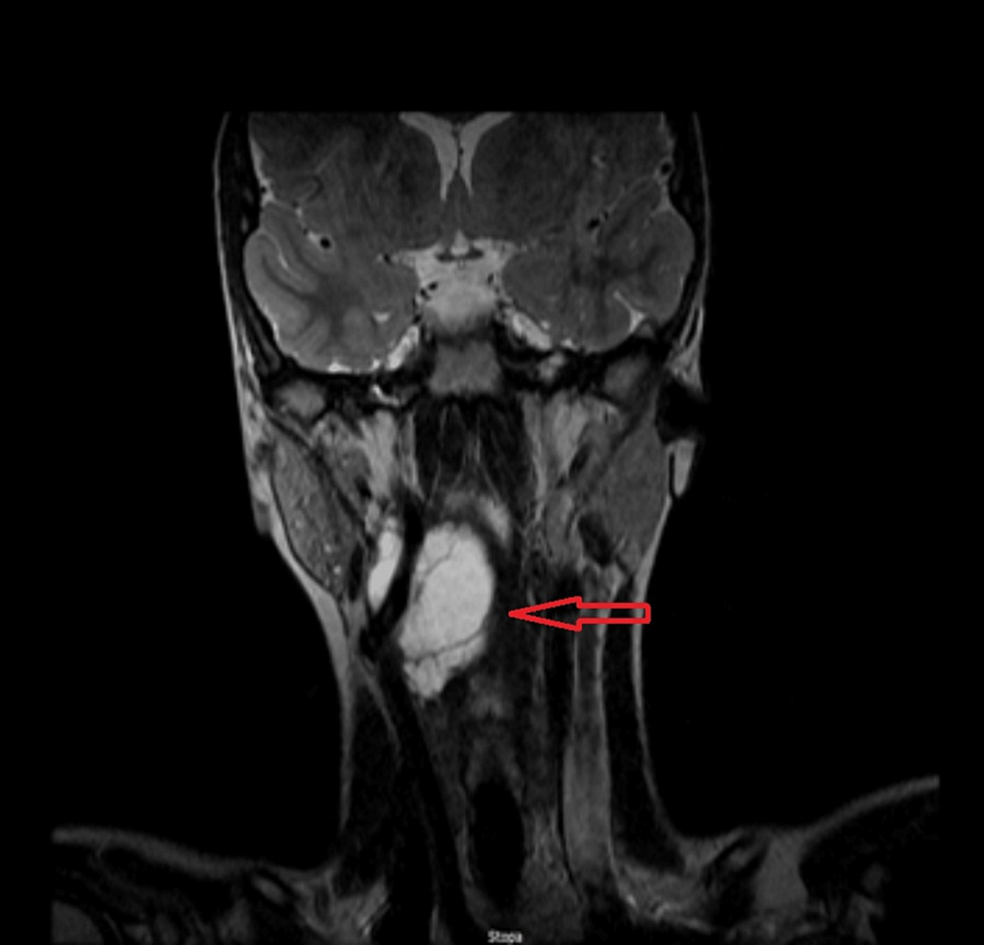
Magnetic resonance imaging, coronal T2-weighted images showing a retropharyngeal lesion extending to the right of the midline and covering the anterior part of the common carotid artery. Red arrow shows a lesion of interest.

Due to suspicion of Chiari's malformation, he underwent magnetic resonance imaging (MRI) of the craniocervical junction. The T2-weighted coronal and sagittal sequences have revealed a 36 × 45 × 13 mm multilocular contrast-enhancing mass in the retropharyngeal space at the C2-C4 level (Figure 1). It deformed the posterior wall of the pharynx, narrowing its lumen and almost clamping the piriform recess. In the lateral part, it was wedged between the internal and common carotid arteries and the internal jugular vein.

**Figure 2:**
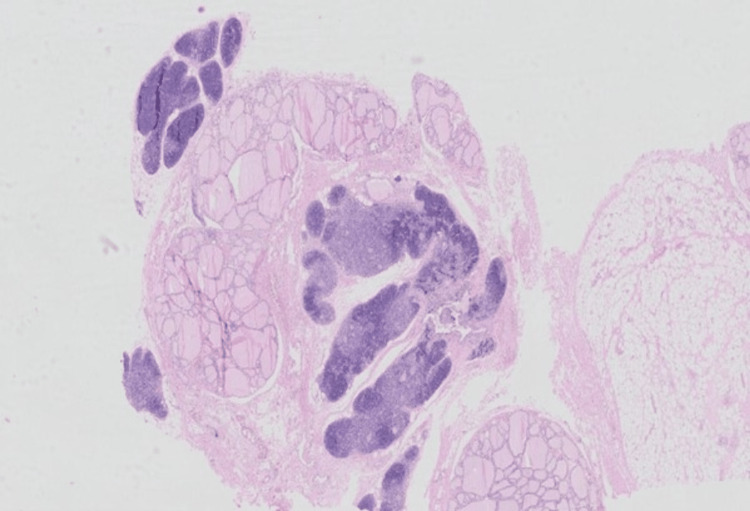
Hematoxylin and eosin staining of thymic tissue.

The patient underwent tumor resection via a lateral submandibular approach. The cyst was totally removed with a fragment of thyroid tissue. Histopathological examination of the specimen revealed ectopic thymic tissue (Figure 2).

**Figure 3:**
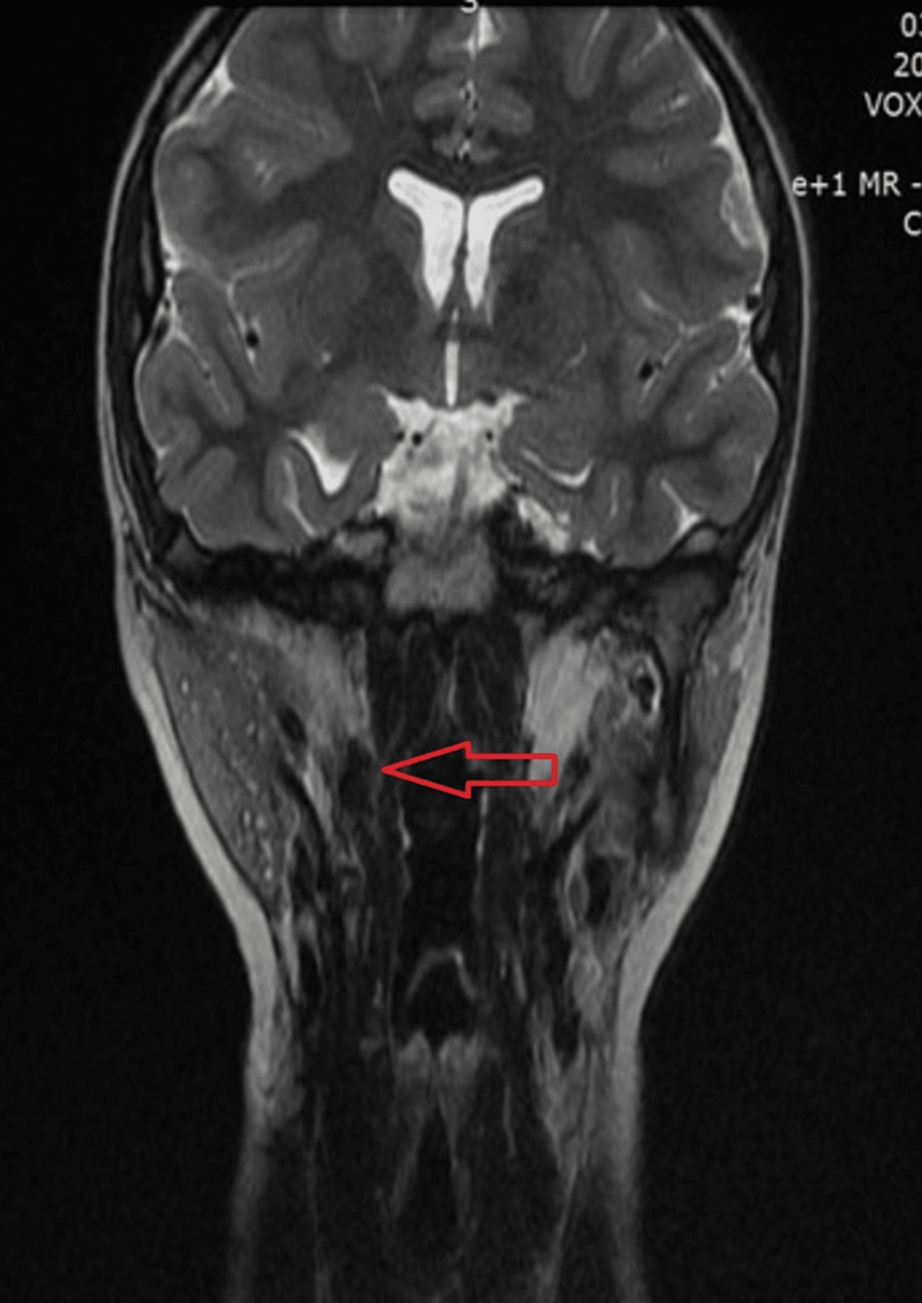
T2-weighted images one year after ectopic thymus resection showing complete removal of the lesion. Red arrow shows the post-operative area.

After one year, the patient underwent a follow-up examination with an MRI of the craniocervical junction, which showed no evidence of residual tissue (Figure 3).

## Discussion

The ectopic thymus is found at autopsy in 1% of cases [6], most commonly between the first and fifth months of life (44%), in which cases the thymus is usually absent in the anterior mediastinum (62%) [7].

Ectopic or accessory thymic tissue can be found anywhere along the pathway of descent: in the adjacency of the superior vena cava, brachiocephalic vessels or the aorta, in the posterior mediastinum, subcutaneous tissue, with the retropharyngeal location being highly unusual [1,8].

Symptoms of an ectopic cervical thymus occur in approximately 10% of cases and include dysphagia, respiratory distress, and recurrent upper respiratory infections [9-11].

The cervical thymus may be found as a mass in the necks of children [9]. It's especially visible and palpable in situations associated with increased intrathoracic pressure (e.g., screaming, crying, deep inspiration, sitting up from a supine position, defecation, etc.) [12]. The ectopic tissue undergoes hyperplasia during the first decade of life, after vaccination or infection [10]. Its presentation in infants and children is determined by its location. It can manifest as a neck mass [9,10], which can cause symptoms of compression of adjacent structures such as the trachea [10] or esophagus [1,10]. There may be other malformations associated with an ectopic thymus, especially cardiovascular (71%), similar to those found in 22q11.2 deletion syndrome [6]. Reported cases of an ectopic thymus in the literature have been surgically removed with no recurrences in the follow-up [12].

Retropharyngeal thymus concurrent with DiGeorge syndrome is an exceptionally rare entity, even relatively to all cases of its ectopia. A search for other similar reports was conducted within the Embase, Pubmed, and ScienceDirect databases using the following formula: (“retropharyngeal” OR “retropharynx”) AND (“thymus” OR “thymic”) AND (“digeorge” OR “22q11” OR “22q11.2”). This, however, yielded no results for the retropharyngeal location specifically.

The human thymus typically stems from the third and possibly fourth pharyngeal pouches [2,3]. Its primordium appears at the beginning of the seventh gestational week as a bilateral predecessor to both the thymic lobe and the ipsilateral inferior parathyroid gland. The dorsal part of this primordium starts to separate as a primitive inferior parathyroid gland, while the ventral portion begins its secession as a primitive thymus. The two then fully disconnect, after which the latter travels ventrally and caudally to eventually fuse with the contralateral lobe at the superior aspect of the pericardium in the mid-eighth gestational week [3].

It is worth noting, however, that this generally accepted theory of thymic embryogenesis does not explain the retropharyngeal location of the pathology presented in this case. As such, two explanations are possible: either the organogenetic process in this patient was somehow adversely affected, which resulted in an aberrant direction of migration of the primordium, or our understanding of the genesis of the thymus is incomplete.

However, puzzling this case may be, an ectopic thymus should not be thought of as a first step in the differential diagnosis of retropharyngeal masses. The usual suspects in this kind of lesion most frequently include neoplasms, abscesses, myxedema, spinal trauma, foreign objects, and lymphadenopathies [13].

## Conclusions

Under normal circumstances, the thymus is an immunoregulatory organ located in the anterosuperior mediastinum. In rare cases, some parts of its tissue or the entire organ can be found along the normal path of its embryological descent and can manifest in clinically relevant forms as an accessory or ectopic thymus. The regular pathway for thymic descent starts in the third (and possibly fourth) laryngeal pouch and proceeds ventrally and caudally. As such, at no point during its formation should it be found behind the pharynx, in the prevertebral area.

The ectopic retropharyngeal thymus, although an unusual finding, let alone in patients diagnosed with 22q11.2 deletion syndrome, needs to be considered as a potential diagnosis for patients presenting with masses located in the retropharyngeal space. Other diagnostic considerations must include such conditions as neoplasms, abscesses, myxedema, spinal trauma, foreign objects, and lymphadenopathies.
